# Astragaloside IV attenuates proteinuria in streptozotocin-induced diabetic nephropathy via the inhibition of endoplasmic reticulum stress

**DOI:** 10.1186/s12882-015-0031-7

**Published:** 2015-03-31

**Authors:** Zeng Si Wang, Fei Xiong, Xiao Hang Xie, Dan Chen, Jian Hua Pan, Li Cheng

**Affiliations:** Department of Nephrology, Wuhan No.1 Hospital, Wuhan, 430000 Hubei Province PR China

**Keywords:** Astragaloside IV, Diabetic nephropathy, Podocytes, Proteinuria, Endoplasmic reticulum stress, Apoptosis

## Abstract

**Background:**

Diabetic nephropathy (DN) is a major cause of Chronic Kidney Disease and End-Stage Renal Disease throughout the world; however, the reversibility of diabetic nephropathy remains controversial. Endoplasmic reticulum (ER) stress plays an important role in the pathogenesis of DN. Astragaloside IV (AS-IV) is derived from *Astragalus membranaceus (Fisch) Bge*, a widely used traditional herbal medicine in China, and has diverse pharmacological activities including the attenuation of podocyte injury and amelioration of proteinuria in idiopathic nephrotic syndrome. The present study aimed to investigate the effect and mechanism of AS-IV on proteinuria in the rat streptozotocin (STZ)-induced model of diabetes.

**Methods:**

Male Sprague–Dawley (SD) rats were randomly divided into four groups: normal control (Normal group), diabetic nephropathy (Model group), diabetic nephropathy plus AS-IV treatment (AS-IV group) and diabetic nephropathy plus 4-phenyl butyric acid treatment (PBA group). ER stress was induced in cultured human podocytes, pretreated with or without AS-IV, with tunicamycin (TM). At the end of 8 weeks, serum creatinine (Scr), blood urea nitrogen (BUN) and 24-hour urinary protein excretion rate (UAER) were determined. Renal morphology was examined after periodic acid-Schiff staining of kidney sections. Apoptosis of podocytes was measured by flow cytometry. The total expression and phosphorylation of eIF2α, PERK and JNK, and the expression of CHOP and cleaved caspase-3 were determined by western blotting. The expression of glucose-regulated protein 78 (GRP78) and 150 kDa oxygen-regulated protein (ORP150) mRNA and protein was determined by real-time PCR and western blotting respectively.

**Results:**

AS-IV treatment significantly reduced urinary albumin excretion, plasma creatinine and blood urea nitrogen levels, and prevented the mesangial matrix expansion and increase in mean mesangial induced by STZ. AS-IV also prevented the phosphorylation of eIF2α, PERK and JNK, and inhibited the expression of GRP78 and ORP150 markedly, both *in vivo* and *in vitro*. AS-IV inhibited the TM-induced apoptosis of podocytes, concomitant with decreased CHOP expression and cleaved caspase-3.

**Conclusions:**

This study supports the hypothesis that AS-IV reduces proteinuria and attenuates diabetes, which is associated with decreased ER stress. This might be an important mechanism in the renoprotective function of AS-IV in the pathogenesis of DN.

## Background

Diabetic nephropathy (DN) is a major cause of chronic kidney disease throughout the world and is the largest cause of end stage of renal disease in the United States [1,2]. Currently, the fundamental therapy for DN is good control of hyperglycemia and BP, and inhibition of the renin-angiotensin-aldosterone system [3,4]. These therapies can be effective in slowing progression but ineffective in reversing established complications. Therefore, it remains necessary to develop new strategies that both slow the progression of the disease and reverse established damage.

The endoplasmic reticulum (ER) plays an important role in the folding and processing of newly synthesized proteins. Pathophysiological stress will lead to the accumulation of misfolded and unfolded proteins, invoking a well-conserved intracellular signaling pathway, known as the unfolded protein response (UPR). There are three canonical arms of the UPR: PKR-like eukaryotic initiation factor 2A kinase (PERK), which rapidly attenuates protein translation; the activating transcription factor-6 (ATF6) and the inositol-requiring enzyme-1 (IRE1α) cascades which transcriptionally upregulate ER chaperone genes that promote proper folding and ER-associated degradation of proteins, allowing the folding machinery of the ER to catch up with the backlog of unfolded proteins. However, excessive and prolonged upregulation of the UPR may lead to cell injury and death.

ER stress plays a key role in cancer, diabetes, and cardiovascular and neurodegenerative diseases [5-8]. Recent experimental studies showed that ER stress is involved in the pathogenesis of diabetes complications, including nephropathy and early neuropathy, and therefore is being recognized as an emerging target for therapy [9-11].

Astragaloside IV (AS-IV) is a small molecular saponin found in *Astragalus membranaceus (Fisch) Bge*, an herb widely used in traditional medicine in China. Recent studies have shown that the molecule has diverse pharmacological activities including anti-inflammatory, anti-hypertensive, anti-diabetes and myocardial protective properties, both *in vitro* and *in vivo* [12-15]. Studies also showed that AS-IV can attenuate podocyte injury and ameliorate proteinuria in adults with idiopathic nephrotic syndrome [16]. The combination of AS-IV with *Angelica sinesis* and ligustrazine not only improved clinical symptoms, increasing serum albumin and lowering blood lipid level, but also reduced urinary protein excretion [17,18]. However, the mechanism by which AS-IV ameliorated proteinuria in DN has not been well defined. In the present study, we investigate the effect of AS-IV on the expression of ER stress signals both in a rat streptozotocin (STZ)-induced model of DN and in podocytes stimulated with tunicamycin. Our study demonstrates that the renoprotective effects of AS-IV are associated with the attenuation of ER stress.

## Methods

### Animals and treatment

All animal procedures were approved by the ethics committee of Huazhong University of Science and Technology and complied with guidance for the Care and Use of Laboratory Animals (National Research Council, National Academy Press, Washington, DC, 1996). Healthy male 6-week-old Sprague–Dawley (SD) rats weighing 180–200 g were kept under pathogen-free conditions. Rats were injected intraperitoneally with 40 mg/kg body weight STZ in 100 mmol/L citrate buffer (pH 4.6) for 5 consecutive days (after an overnight fast). Rats with a blood glucose levels over 300 mg/dL were considered as having diabetes [19], and were treated with AS-IV (10 mg/kg/day) or PBA (1 g/kg/day) 2 weeks after STZ injections. Drugs were administered via oral gavage to the rats for 8 weeks. The normal control and diabetic control rats received an equal volume of vehicle. Rats were kept in individual metabolic cages for 24 h urine collection at the end of treatment. Urine was centrifuged at 800 × *g* for 10 min at 25°C prior to testing. At the end of 8 weeks of treatment, rats were anesthetized with pentobarbital sodium and blood samples taken from the abdominal aorta, and then centrifuged for 15 min at 2000 × *g* to obtain plasma for measuring biochemical parameters. Whole urine and plasma was stored at −70°C and thawed just before use. Animals were then sacrificed and half of each kidney was snap-frozen in liquid nitrogen for RNA preparation/protein extraction, and the other half was processed for histology and immunostaining.

### Urine and plasma measurements

Urinary albumin concentrations were measured using an ELISA Kit (Nanjing Jiancheng Bioengineering Institute, Nanjing, China) according to the manufacturer’s instructions. Blood urea nitrogen, creatinine and urine creatinine were measured using an automatic biochemistry analyzer (Hitachi Model 7600, Hitachi High-Technologies, Tokyo, Japan).

### Human podocyte culture and treatment

Human podocytes were cultured and differentiated in RPMI culture medium containing 10% FBS and 1% penicillin/streptomycin as previously described [20]. In brief, immortalized normal human podocytes were propagated at 33°C and then thermoshifted for differentiation for 14 days at 37°C. Terminally differentiated podocytes were serum and insulin starved in 0.2% FBS for the experiments. After pretreatment with AS-IV (100 μg/ml) or PBA (10 mmol/L), podocytes were treated with 5 μg/mL tunicamycin to induce ER stress [21,22].

### Apoptosis assay

Apoptosis was assessed by annexin V-FITC and PI staining followed by analysis by flow cytometry (Beckman-Coulter, Indianapolis, IN, USA). Briefly, after the indicated treatment, the cells were harvested by trypsinization, fixed with 70% (v/v) alcohol at 4°C for 30 min and washed with PBS. After centrifugation, cells were incubated in 0.1 mL of phosphate-citric acid buffer (0.2 mol/L Na_2_HPO_4_, 0.1 mol/L citric acid, pH 7.8) for 30 min at room temperature. The cells were centrifuged and resuspended in 0.5 mL PI solution containing Triton X-100 (0.1% v/v), RNase (100 μg/mL) and PI (80 μg/mL), then analyzed by flow cytometry.

### Renal histopathology

Kidneys were rapidly dissected, fixed overnight in 10% buffered formalin, embedded in paraffin, and 4-mm sections subjected to periodic acid-Schiff (PAS) staining. The ratio of mesangial matrix area relative to total glomerular area was quantified in 20 glomeruli from each group using Image-Pro Plus 6.0 software (Media Cybernetics, Bethesda, MD, USA), as described previously [23].

### Quantitative real-time PCR

Total RNA was extracted from kidneys or podocytes using TRIzol regent (Invitrogen, Carlsbad, CA, USA) according to the manufacturer’s instructions. cDNA from total RNA was synthesized by reverse-transcription reaction using a ThermoScript RT-PCR system (Toyobo, Osaka, Japan). The primers for GRP78, ORP150 and actin were synthesized by Sangon Biotechnology Company (Shanghai, China). Real-time PCR analysis was performed in a final volume of 25 μL containing 12.5 μL SYBR Green I, using a LightCycler instrument (Roche Diagnostic, Mannheim, Germany). The following thermal cycling profile for PCR was used: one cycle at 95°C for 30 s, followed by 40 cycles at 95°C for 5 s, and at 58°C for 5 s, with a final extension step at 72°C for 30 min. PCR products were analyzed by melting curve to confirm correct amplification. The fold-change analysis was normalized based on RNA levels of actin in the same sample. The following primers were used: R-ORP150-F, GATCACCGTGCCAGCCTTTT; R-ORP150-R, CCTCCTTAGTCTTCACCGTTTG; R-GRP78-F, TCGTATGTGGCCTTCACTCC; R-GRP78-R, TTCTTCTGGGGCAAATGTCT; R-actin-F, CGTTGACATCCGTAAAGACCTC; R-actin-R, TAGGAGCCAGGGCAGTAATCT.

### Western blotting

Total protein concentration was measured using the BCA method, and 50 μg protein per lane was separated by SDS-Polyacrylamide Gel Electrophoresis (SDS-PAGE) on 8% or 10% polyacrylamide gels and transferred to poly vinylidene fluoride (PVDF) membranes. Membranes were incubated with primary antibodies for either 2 h at room temperature or overnight at 4°C. The following antibodies were used (at a 1:1,000 dilution unless otherwise indicated): total eIF2α (#5324; Cell Signaling Technology (CST), Danvers, MA, USA), phospho-eIF2α (#3398; CST), total PERK (#20582-1-AP; Proteintech Group, Chicago, IL, USA), phospho-PERK (sc-32577: Santa Cruz, Dallas, TX, USA), total JNK (#9258; CST), phospho-JNK (#4668; CST), GRP78 (#3216-1; Epitomics, Burlingame, CA, USA), ORP150 (#3905-1; Epitomics), CHOP (sc-575; Santa Cruz) and cleaved caspase-3 (#9661; CST). Equal loading of protein between lanes was confirmed by subsequent immunoblotting of β-actin (1:5,000; Sigma, St Louis, MO, USA). After incubation with horseradish peroxidase-conjugated goat anti-mouse or donkey anti-rabbit antibody (1:5,000; Jackson ImmunoResearch Laboratories, West Grove, PA, USA) for 1 h at room temperature, immunodetection was performed by chemiluminescence.

### Statistical analysis

All statistical analyses were performed using SPSS 15.0 software (IBM Corp., Chicago IL). Data are expressed as the means ± SD. One-way ANOVA was used to determine the significance of differences between groups. A *P*-value of *P* < 0.05 was considered statistically significant.

## Results

### Characteristics of experimental rats

Table 1 shows the clinical and laboratory characteristics of the four groups of rats at the end of the experimental period. Compared with the Normal group, the rats injected with STZ to induce diabetes (Model group) showed significantly higher right kidney weight, kidney/body weight ratio and glucose levels, and greater impairment of kidney function. After 8 weeks of treatment with AS-IV (AS-IV group), the urinary albumin excretion, plasma creatinine and blood urea nitrogen levels were markedly reduced; however, there were no differences in body weight and blood glucose between the Model group and AS-IV treated group. PBA, a chemical chaperone approved for use in renal disease, showed similar effects to AS-IV. However, we demonstrated that PBA additionally lowered blood glucose levels, which was different to AS-IV. This therefore suggests that AS-IV attenuates the functional abnormalities present in diabetic nephropathy independently of any hypoglycemic effect.

**Table 1:** **Body weight, kidney weight, serum and urinary parameters in different groups after 8 weeks of treatment**

	**Normal**	**Model**	**AS-IV**	**PBA**
Body weight (g)	240.3 ± 14.3	225.6 ± 11.5^△^	226.4 ± 8.4	230.7 ± 12.0*
Kidney weight (g)	1.4 ± 0.1	1.6 ± 0.4^△^	1.4 ± 0.1*	1.4 ± 0.2*
Kidney/body weight ratio (%)	5.7 ± 0.5	7.1 ± 1.0^△^	6.3 ± 0.4*	6.2 ± 0.5*
SCr (μmol/L)	25.5 ± 10.8	51.0 ± 11.5^△^	33.4 ± 8.7*	27.6 ± 6.9*
BUN (mmol/L)	15.4 ± 3.9	53.8 ± 11.8^△^	26.1 ± 2.3*	25.4 ± 2.5*
Urinary albumin excretion (μg/day)	571.7 ± 72.4	1510.0 ± 392.7^△^	799.4 ± 121.5*	757.9 ± 105.5*
Blood glucose (mmol/L)	8. 5 ± 0.9	21.9 ± 5.4^△^	19.8 ± 1.2	12.3 ± 4.9*

Data are presented as the mean ± SD. ^△^ *P* < 0.05 *vs* Normal, *P < 0.05 *vs* Model.

### Changes in kidney morphology

**Figure 1:**
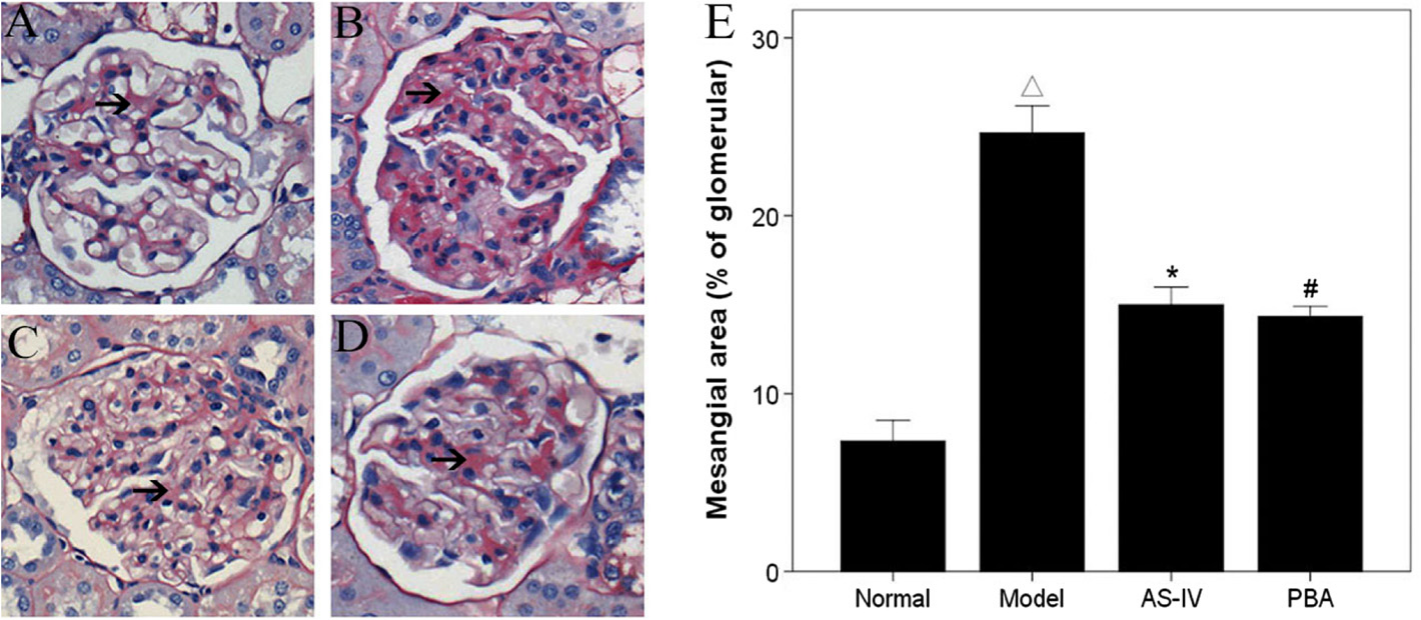
**AS-IV attenuates mesangial matrix expansion in a rat model of diabetic nephropathy. A**–**D**: Representative images of PAS stained kidney tissue (×400). **A**, Normal group; **B**, Model group; **C**, AS-IV group; **D**, PBA group. **E**: The ratios of mesangial area to total glomerular area were averaged from 30 glomeruli per rat. Data are presented as the mean ± SD. ^△^ *P* < 0.05 *vs.* Normal, *P < 0.05 *vs.* Model.

Figure 1 shows representative photomicrographs of mesangial matrix accumulation in PAS-stained kidneys from the four groups. Mesangial area (black arrows) was assessed in PAS-stained sections and expressed as a percentage of the glomerular area. Eight weeks after STZ injection, the diabetic rats demonstrated mesangial matrix expansion in glomeruli compared with normal control rats. The mesangial area was also significantly increased in diabetic rats compared with normal rats. In contrast, AS-IV treated rats showed significantly attenuated mesangial cell proliferation and mesangial expansion compared with the untreated diabetic rats.

### AS-IV alleviates ER stress

**Figure 2:**
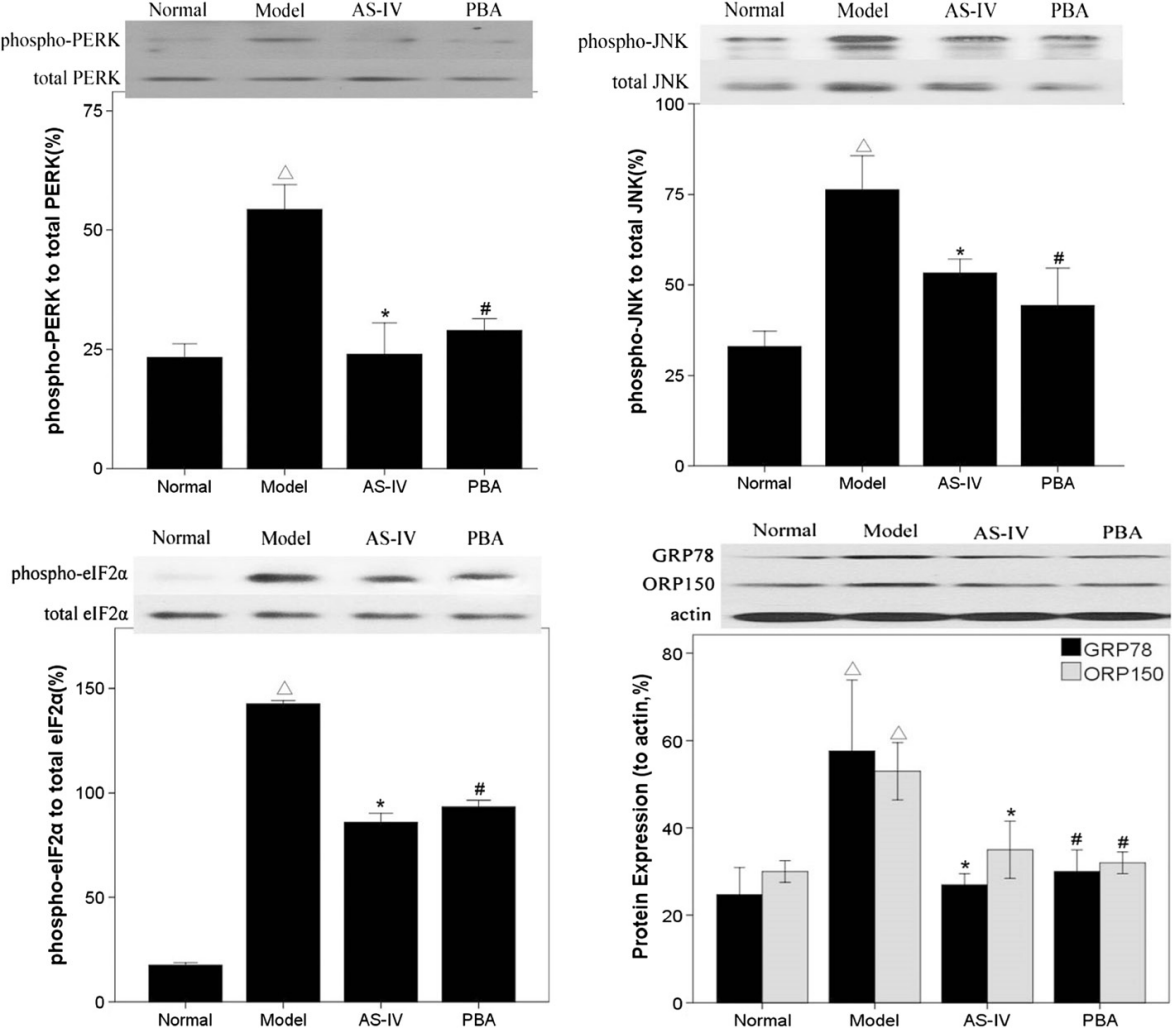
**AS-IV alleviates ER stress in rats with STZ-induced DN.** Representative western blot analyses of total and phosphorylated PERK, eIF2a and JNK and total expression of GRP78 and ORP150. Data are presented as the mean ± SD, n = 6 per group. ^△^ *P* < 0.05 *vs.* Normal, *P < 0.05 *vs.* Model.

**Figure 3:**
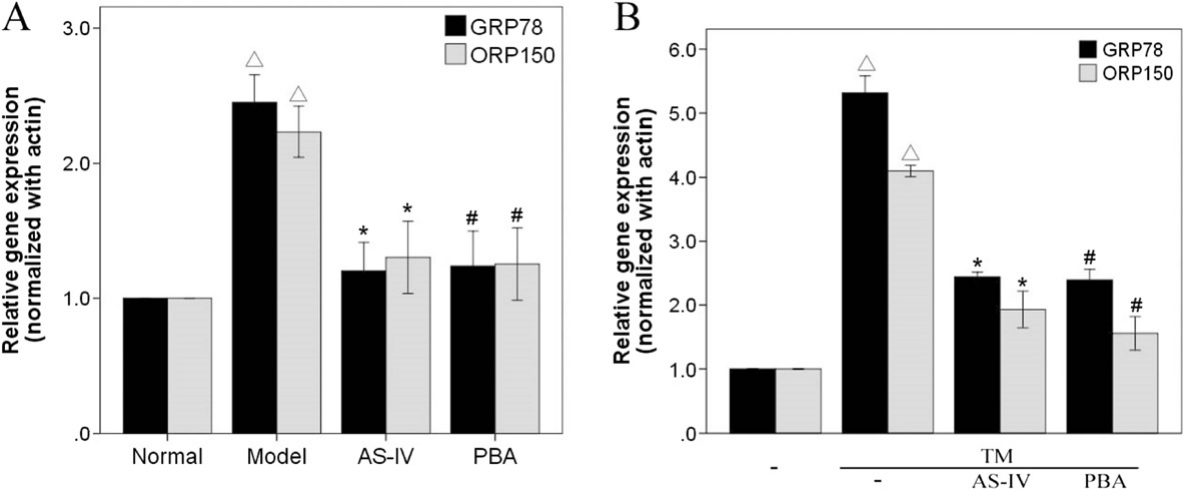
**AS-IV decreases the expression of GRP78 and ORP150.** The expression of GRP78 and ORP150 mRNA in rats injected with STZ to induce diabetes **(A)**. The expression of GRP78 and ORP150 mRNA in podocytes treated with TM **(B)**. Data are presented as the mean ± SD, n = 6 per group. ^△^ *P* < 0.05 *vs.* Normal, *P < 0.05 *vs.* Model.

Compared with normal rats, the phosphorylation of PERK, eIF2α and JNK were upregulated in diabetic rats, while total PERK, eIF2α and JNK expression was unchanged. Furthermore, GRP78 and ORP150 were increased, at both the mRNA and protein levels. These results indicate the ER stress was activated in glomeruli of diabetic rats (Figures 2 and 3A). PBA is a low molecular weight fatty acid that restores ER function, and our study confirmed that the UPR was blocked after treatment with PBA. Interestingly, after being treated with AS-IV for 8 weeks, the activation of the UPR in rats was blunted, and the phosphorylation level of PERK, eIF2α and JNK level were reduced compared with untreated rats. AS-IV also reduced the expression of GRP78 and ORP150, at both the mRNA and protein level. Similar results were found with PBA treatment.

**Figure 4:**
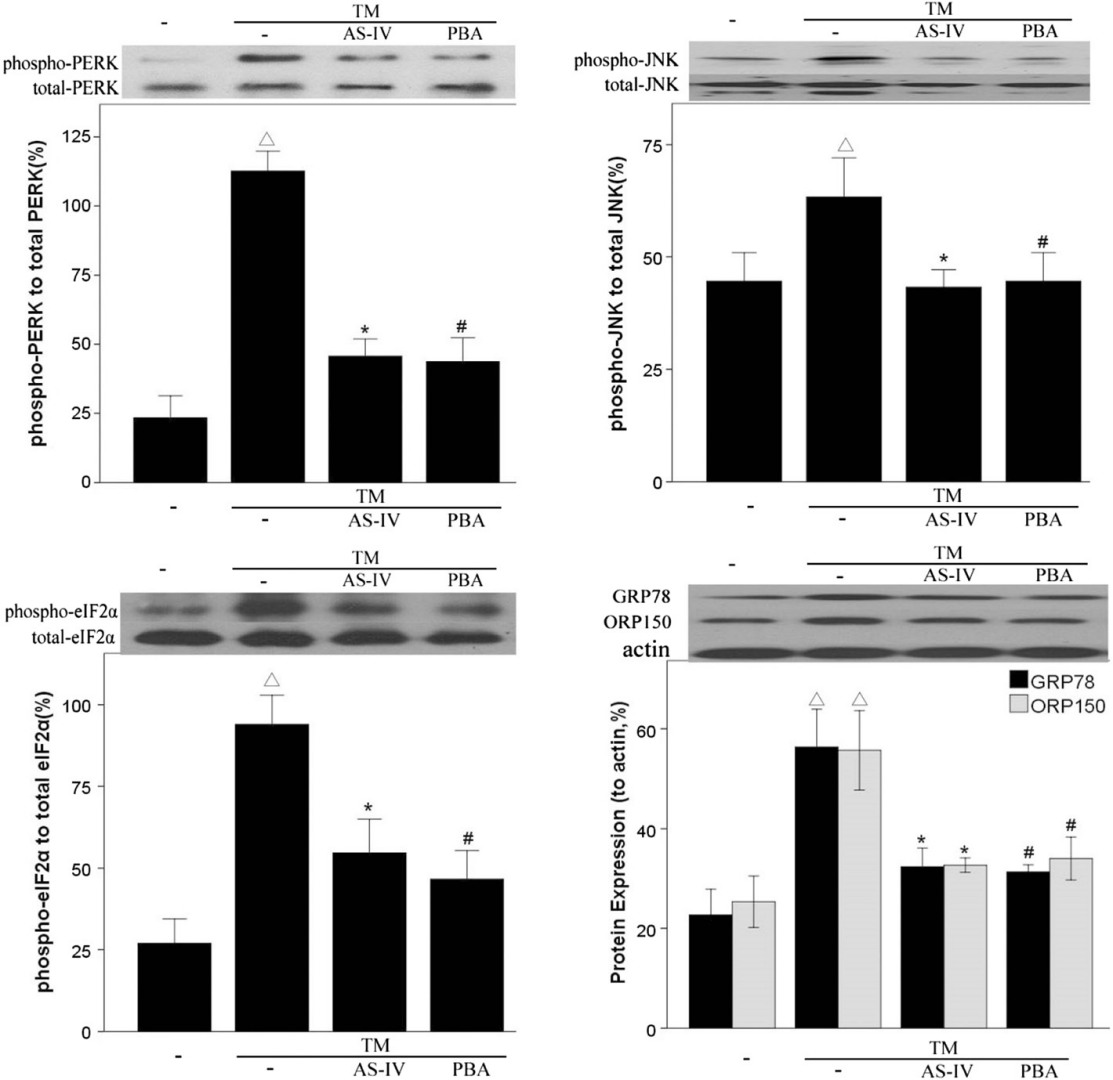
**Astragaloside IV alleviates ER stress in podocytes treated with TM.** Representative western blot analyses of total and phosphorylated PERK, eIF2a and JNK and expression of GRP78 and ORP150. Data are presented as the mean ± SD, n = 6 per group. ^△^ *P* < 0.05 *vs.* Untreated, *P < 0.05 *vs.* TM treated.

Tunicamycin is an inhibitor of glycosylation, which causes retention of unfolded proteins in the ER, and is widely used as an inducer of ER stress [21]. We therefore examined the effects of AS-IV on TM-induced ER stress in podocytes (Figures 3B and 4). Our results showed that the expression of ORP150 and GRP78 were increased, and phosphorylation of PERK, eIF2α and JNK were upregulated in podocytes treated with TM, indicating that TM did induce ER stress in podocytes. However, the induction of ER stress by TM was blocked when cells were pretreated with AS-IV or PBA. Our findings demonstrated that ORP150 and GRP78 were inhibited both at the mRNA and protein levels when cells were treated with AS-IV, while simultaneously, the phosphorylation of PERK, eIF2α and JNK were downregulated. Therefore, AS-IV inhibits TM-induced ER stress in podocytes.

### AS-IV reduces TM-induced apoptosis in podocytes

**Figure 5:**
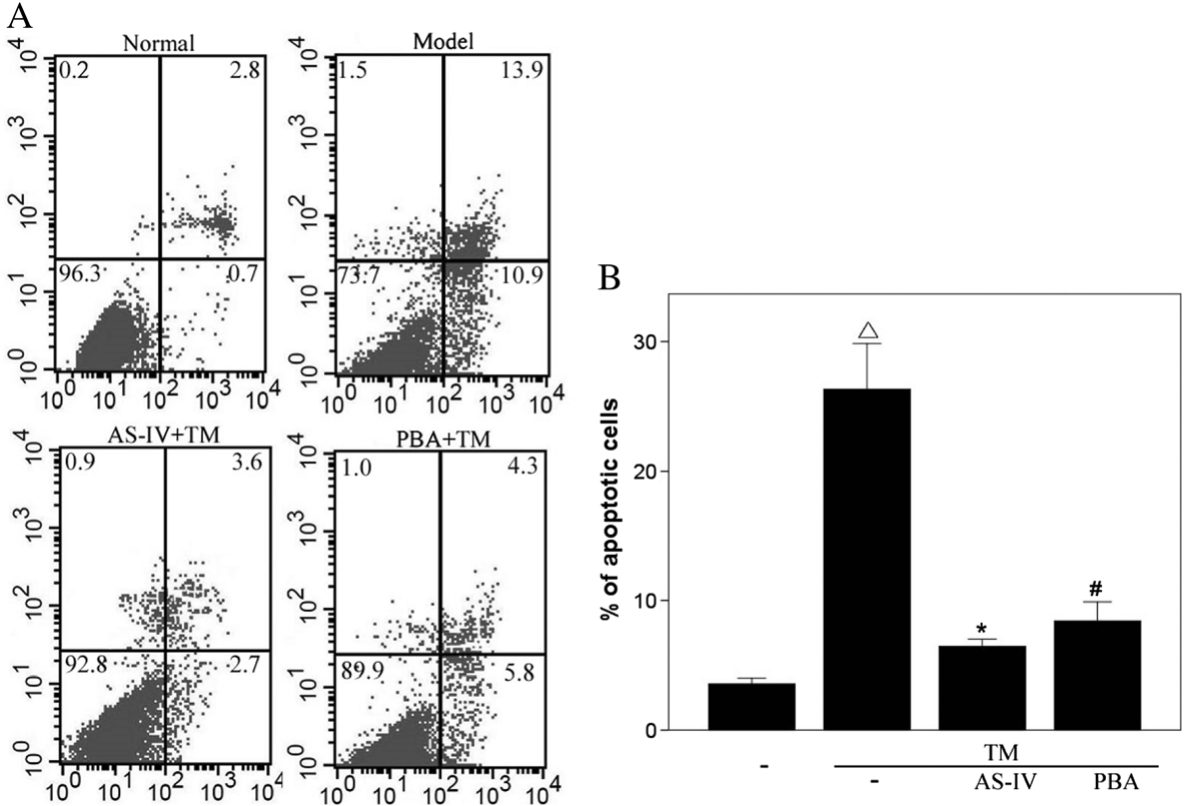
**AS-IV attenuates apoptosis of podocytes induced by TM.** Podocyte apoptosis was induced with tunicamycin (TM), after pretreatment with or without AS-IV, and then cells were co-stained with propidium iodide (PI) and annexin V-FITC (A-V) followed by flow cytometric analysis. Apoptotic cells refer to percentage of cells encompassing both A-V single positive and A-V/PI double-positive cells. Representative scatter plots are shown in Figure 5 **A**. The percentage of apoptotic cells determined by flow cytometry are shown in Figure 5 **B**. (n = 6 for both experiments). Data are presented as the mean ± SD. ^△^ *P* < 0.05 *vs*. Untreated, *P < 0.05 *vs*. TM treated.

The TM-induced apoptosis of podocytes was confirmed by flow cytometry after co-staining cells with annexin V and propidium iodide (Figure 5). Compared with the untreated control, apoptosis was induced when podocytes were treated with TM. Our data showed that AS-IV had a similar anti-apoptotic effect to PBA; it not only decreased early apoptotic events (located in the lower right quadrant of the flow cytometry diagrams), but also reduced the number of cells undergoing late apoptosis (in the upper right quadrant).

**Figure 6:**
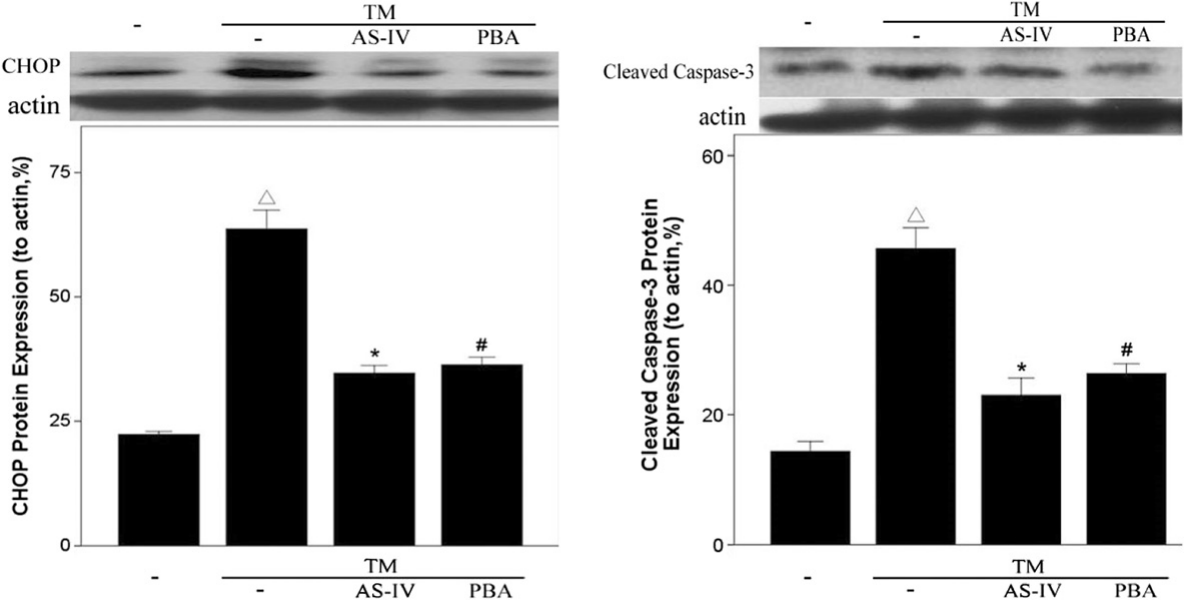
**AS-IV inhibits the expression of CHOP and cleaved of caspase-3 in podocytes stimulated with TM.** Data are presented as the mean ± SD. ^△^ *P* < 0.05 *vs.* Untreated, *P < 0.05 *vs.* TM treated.

Studies have demonstrated the critical role of CHOP in ER stress-induced cell death [24]. Consistent with this notion, TM treatment resulted in a significant increase in CHOP expression and cleaved caspase-3, whereas AS-IV treatment significantly attenuated these changes, as shown in Figure 6. Collectively, these data support the notion that AS-IV prevents TM-induced podocyte apoptosis partly through the attenuation of CHOP induction, inhibiting caspase-3 activation.

## Discussion

DN is the most common cause of chronic kidney disease [25]. The early abnormalities of DN are glomerular hyperfiltration, increased renal albumin permeability followed by the development of glomerular mesangial cell proliferation, extracellular matrix accumulation and glomerulosclerosis. In this study we demonstrated that AS-IV reduced the urinary albumin excretion, reversed the deterioration of renal function, prevented the mesangial matrix expansion and increase in mean mesangial area in a STZ-induced model of DN. AS-IV also inhibited podocyte apoptosis induced by TM. However, as AS-IV showed no effect on blood glucose levels, we suggest that the renoprotective effect of AS-IV is related ER stress inhibition, independent of any hypoglycemic effect.

Persistent proteinuria is a strong prognostic indicator for progression of DN. Furthermore, proteinuria and hyperglycemia generate reactive oxygen species and require a marked increase in the synthesis of membrane proteins in the kidney, which may result in increased ER stress [26]. Furthermore, accumulating evidence indicates that ER stress contributes to glomerular diseases with proteinuria [27]. Studies also show that both the PERK/eIF2α and IRE1/JNK pathways are activated in DN [11]. Chemical chaperones, which reduce misfolded proteins and thereby mitigate ER stress, have been shown to ameliorate STZ-induced DN [28]. In our study we found that AS-IV significantly alleviated ER stress, both *in vivo* and *in vitro*, by blocking the activation of both the PERK/eIF2α and IRE1/JNK pathways.

GRP78, also referred to as BiP, is a central regulator of ER homeostasis and is involved in the activation of the ER stress response. The 150 kDa oxygen-regulated protein (ORP150) has a pivotal role in maintaining cell viability in response to stress, and stimuli which induce cellular perturbation via the ER often involve ORP150 [29,30]. Therefore, both GRP78 and ORP150 are markers of ER stress. Studies from human kidney biopsies found that renal diseases including minimal change disease (MCD), focal and segmental glomerulosclerosis (FSGS), membranous nephropathy and DN were associated with increased podocyte ORP150/HYOU1 and GRP78 expression. In this study, we confirmed that AS-IV downregulated GRP78 and ORP150 expression, at both the gene and protein level. Therefore, in view of the important role of ER stress in the pathology of kidney impairment, we hypothesize that the renoprotective effect of AS-IV is associated with its biological effect of attenuating ER stress.

Diabetic glomerulosclerosis is defined by an increase in glomerular extracellular matrix, which is mainly synthesized by mesangial cells, leading to renal dysfunction in patients with diabetes [31]. Our research showed that AS-IV significantly decreased mesangial expansion and deposition of extracellular matrix in diabetic rats, suggesting that AS-IV may also attenuates ECM accumulation in DN. The podocyte plays a crucial role in the maintenance of the permeability properties of the glomerular filtration barrier, whereas injury and loss of podocytes will contribute to proteinuria and glomerulosclerosis [32,33]. Studies have shown that apoptosis of podocytes induced by ER stress plays an important role in the process of glomerulosclerosis, and that CHOP (C/EBP homologous protein) is a key mediator of cell death in response to ER stress [34-36]. In the present study, we showed that AS-IV significantly prevented the apoptosis of podocytes induced by TM. We also found that AS-IV attenuated apoptosis partly through the decreased expression of CHOP and inhibition of caspase-3 activation. This provides ample evidence for the hypothesis that the beneficial effect of AS-IV on the progression of DN may related to its attenuation of ER stress.

## Conclusions

In summary, this study indicates that AS-IV can ameliorate the structural and functional abnormalities present in a STZ-induced rat model of DN, with the renoprotective activity mediated through the inhibition ER stress. This novel finding provides support for alternative therapies for the treatment of DN based on targeting the regulation of the ER stress response.

## References

[1] de Boer IH, Rue TC, Hall YN, Heagerty PJ, Weiss NS, Himmelfarb J (2011). Temporal trends in the prevalence of diabetic kidney disease in the United States. JAMA.

[2] Collins AJ, Foley RN, Chavers B, Gilbertson D, Herzog C, Johansen K (2012). 'United states renal data system 2011 annual data report: atlas of chronic kidney disease & end-stage renal disease in the United States. Am J Kidney Dis.

[3] National Kidney F (2012). KDOQI clinical practice guideline for diabetes and CKD: 2012 update. Am J Kidney Dis.

[4] Nelson RG, Tuttle KR (2010). Prevention of diabetic kidney disease: negative clinical trials with renin-angiotensin system inhibitors. Am J Kidney Dis.

[5] McAlpine CS, Werstuck GH (2013). The development and progression of atherosclerosis: evidence supporting a role for endoplasmic reticulum (ER) stress signaling. Cardiovasc Hematol Disord Drug Targets.

[6] Saxena S, Cabuy E, Caroni P (2009). A role for motoneuron subtype-selective ER stress in disease manifestations of FALS mice. Nat Neurosci.

[7] Schonthal AH (2012). Targeting endoplasmic reticulum stress for cancer therapy. Front Biosci.

[8] McKimpson WM, Weinberger J, Czerski L, Zheng M, Crow MT, Pessin JE (2013). The apoptosis inhibitor ARC alleviates the ER stress response to promote beta-cell survival. Diabetes.

[9] Lupachyk S, Watcho P, Stavniichuk R, Shevalye H, Obrosova IG (2013). Endoplasmic reticulum stress plays a key role in the pathogenesis of diabetic peripheral neuropathy. Diabetes.

[10] Lhotak S, Sood S, Brimble E, Carlisle RE, Colgan SM, Mazzetti A (2012). ER stress contributes to renal proximal tubule injury by increasing SREBP-2-mediated lipid accumulation and apoptotic cell death. Am J Physiol Renal Physiol.

[11] Cunard R, Sharma K (2011). The endoplasmic reticulum stress response and diabetic kidney disease. Am J Physiol Renal Physiol.

[12] Luo HM, Dai RH, Li Y (1995). [Nuclear cardiology study on effective ingredients of Astragalus membranaceus in treating heart failure]. Zhongguo Zhong Xi Yi Jie He Za Zhi.

[13] He Y, Du M, Gao Y, Liu H, Wang H, Wu X (2013). Astragaloside IV attenuates experimental autoimmune encephalomyelitis of mice by counteracting oxidative stress at multiple levels. PLoS One.

[14] Zhang N, Wang XH, Mao SL, Zhao F (2011). Astragaloside IV improves metabolic syndrome and endothelium dysfunction in fructose-fed rats. Molecules.

[15] Gui D, Huang J, Guo Y, Chen J, Chen Y, Xiao W (2013). Astragaloside IV ameliorates renal injury in streptozotocin-induced diabetic rats through inhibiting NF-kappaB-mediated inflammatory genes expression. Cytokine.

[16] Zheng R, Deng Y, Chen Y, Fan J, Zhang M, Zhong Y (2012). Astragaloside IV attenuates complement membranous attack complex induced podocyte injury through the MAPK pathway. Phytother Res.

[17] Chen Y, Deng Y, Ni Z, Chen N, Chen X, Shi W (2013). Efficacy and safety of traditional chinese medicine (Shenqi particle) for patients with idiopathic membranous nephropathy: a multicenter randomized controlled clinical trial. Am J Kidney Dis.

[18] Zhong Y, Deng Y, Chen Y, Chuang PY, Cijiang He J (2013). Therapeutic use of traditional Chinese herbal medications for chronic kidney diseases. Kidney Int.

[19] Pichaiwong W, Hudkins KL, Wietecha T, Nguyen TQ, Tachaudomdach C, Li W (2013). Reversibility of structural and functional damage in a model of advanced diabetic nephropathy. JASN.

[20] Saleem MA, O'Hare MJ, Reiser J, Coward RJ, Inward CD, Farren T (2002). A conditionally immortalized human podocyte cell line demonstrating nephrin and podocin expression. JASN.

[21] Inagi R, Nangaku M, Onogi H, Ueyama H, Kitao Y, Nakazato K (2005). Involvement of endoplasmic reticulum (ER) stress in podocyte injury induced by excessive protein accumulation. Kidney Int.

[22] Yan K, Khoshnoodi J, Ruotsalainen V, Tryggvason K (2002). N-linked glycosylation is critical for the plasma membrane localization of nephrin. J Am Soc Nephrol.

[23] Watson AM, Gray SP, Jiaze L, Soro-Paavonen A, Wong B, Cooper ME (2012). Alagebrium reduces glomerular fibrogenesis and inflammation beyond preventing RAGE activation in diabetic apolipoprotein E knockout mice. Diabetes.

[24] Qi Y, Xia P (2012). Cellular inhibitor of apoptosis protein-1 (cIAP1) plays a critical role in beta-cell survival under endoplasmic reticulum stress: promoting ubiquitination and degradation of C/EBP homologous protein (CHOP). J Biol Chem.

[25] Atkins RC, Zimmet P, World Kidney Day Steering C (2010). Diabetic kidney disease: act now or pay later. JASH.

[26] Lindenmeyer MT, Rastaldi MP, Ikehata M, Neusser MA, Kretzler M, Cohen CD (2008). Proteinuria and hyperglycemia induce endoplasmic reticulum stress. J Am Soc Nephrol.

[27] Cybulsky AV (2010). Endoplasmic reticulum stress in proteinuric kidney disease. Kidney Int.

[28] Qi W, Mu J, Luo ZF, Zeng W, Guo YH, Pang Q (2011). Attenuation of diabetic nephropathy in diabetes rats induced by streptozotocin by regulating the endoplasmic reticulum stress inflammatory response. Metab Clin Exp.

[29] Zhang LH, Zhang X (2010). Roles of GRP78 in physiology and cancer. J Cell Biochem.

[30] Arrington DD, Schnellmann RG (2008). Targeting of the molecular chaperone oxygen-regulated protein 150 (ORP150) to mitochondria and its induction by cellular stress. Am J Physiol Cell Physiol.

[31] Tervaert TW, Mooyaart AL, Amann K, Cohen AH, Cook HT, Drachenberg CB (2010). Pathologic classification of diabetic nephropathy. J Am Soc Nephrol.

[32] Daehn I, Casalena G, Zhang T, Shi S, Fenninger F, Barasch N (2014). Endothelial mitochondrial oxidative stress determines podocyte depletion in segmental glomerulosclerosis. J Clin Invest.

[33] Canaud G, Bienaime F, Viau A, Treins C, Baron W, Nguyen C (2013). AKT2 is essential to maintain podocyte viability and function during chronic kidney disease. Nat Med.

[34] Wu J, Zhang R, Torreggiani M, Ting A, Xiong H, Striker GE (2010). Induction of diabetes in aged C57B6 mice results in severe nephropathy: an association with oxidative stress, endoplasmic reticulum stress, and inflammation. Am j pathol.

[35] Sieber J, Lindenmeyer MT, Kampe K, Campbell KN, Cohen CD, Hopfer H (2010). Regulation of podocyte survival and endoplasmic reticulum stress by fatty acids. Am J Physiol Renal Physiol.

[36] Fang L, Li X, Luo Y, He W, Dai C, Yang J (2014). Autophagy inhibition induces podocyte apoptosis by activating the pro-apoptotic pathway of endoplasmic reticulum stress. Exp Cell Res.

